# MRI Asymmetry Index of Hippocampal Subfields Increases Through the Continuum From the Mild Cognitive Impairment to the Alzheimer's Disease

**DOI:** 10.3389/fnins.2018.00576

**Published:** 2018-08-21

**Authors:** Alessia Sarica, Roberta Vasta, Fabiana Novellino, Maria Grazia Vaccaro, Antonio Cerasa, Aldo Quattrone, The Alzheimer's Disease Neuroimaging Initiative

**Affiliations:** ^1^Neuroscience Centre, Magna Graecia University, Catanzaro, Italy; ^2^Neuroimaging Research Unit, Institute of Molecular Bioimaging and Physiology, National Research Council, Catanzaro, Italy; ^3^S. Anna Institute and Research in Advanced Neurorehabilitation, Crotone, Italy

**Keywords:** hippocampus, hippocampal subfields, asymmetry index, Alzheimer's disease, Mild Cognitive Impairment, neuroimaging

## Abstract

**Objective:** It is well-known that the hippocampus presents significant asymmetry in Alzheimer's disease (AD) and that difference in volumes between left and right exists and varies with disease progression. However, few works investigated whether the asymmetry degree of subfields of hippocampus changes through the continuum from Mild Cognitive Impairment (MCI) to AD. Thus, aim of the present work was to evaluate the Asymmetry Index (AI) of hippocampal substructures as possible MRI biomarkers of Dementia. Moreover, we aimed to assess whether the subfields presented peculiar differences between left and right hemispheres. We also investigated the relationship between the asymmetry magnitude in hippocampal subfields and the decline of verbal memory as assessed by Rey's auditory verbal learning test (RAVLT).

**Methods:** Four-hundred subjects were selected from ADNI, equally divided into healthy controls (HC), AD, stable MCI (sMCI), and progressive MCI (pMCI). The structural baseline T1s were processed with FreeSurfer 6.0 and volumes of whole hippocampus (WH) and 12 subfields were extracted. The AI was calculated as: (|Left-Right|/(Left+Right))^*^100. ANCOVA was used for evaluating AI differences between diagnoses, while paired *t*-test was applied for assessing changes between left and right volumes, separately for each group. Partial correlation was performed for exploring relationship between RAVLT summary scores (Immediate, Learning, Forgetting, Percent Forgetting) and hippocampal substructures AI. The statistical threshold was Bonferroni corrected *p* < 0.05/13 = 0.0038.

**Results:** We found a general trend of increased degree of asymmetry with increasing severity of diagnosis. Indeed, AD presented the higher magnitude of asymmetry compared with HC, sMCI and pMCI, in the WH (AI mean 5.13 ± 4.29 SD) and in each of its twelve subfields. Moreover, we found in AD a significant negative correlation (*r* = −0.33, *p* = 0.00065) between the AI of parasubiculum (mean 12.70 ± 9.59 SD) and the RAVLT Learning score (mean 1.70 ± 1.62 SD).

**Conclusions:** Our findings showed that hippocampal subfields AI varies differently among the four groups HC, sMCI, pMCI, and AD. Moreover, we found—for the first time—that hippocampal substructures had different sub-patterns of lateralization compared with the whole hippocampus. Importantly, the severity in learning rate was correlated with pathological high degree of asymmetry in parasubiculum of AD patients.

## Introduction

Brain hemispheric specialization is among the central features of functional cortical organization in humans (Goldberg et al., 2013) and less lateralization is usually associated with better cognitive ability (Catani et al., 2007). The morphological asymmetries of brain regions were traditionally correlated to the optimal information processing, language function, visuospatial task, attention, and many aspects of emotion (Toga and Thompson, 2003; Kim et al., 2012; Woolard and Heckers, 2012) in normal population (Guadalupe et al., 2014). The laterality of human brain varies with the aging (Long et al., 2013; Lucarelli et al., 2013) and moreover, changes in the normal pattern of asymmetry could be representative of a brain pathology and could serve as a neuroanatomical marker or as a risk factor (Toga and Thompson, 2003; Woolard and Heckers, 2012; Okada et al., 2016). In other words, the existence of asymmetry in brain regions where the symmetry is expected or, on the contrary, the absence of asymmetry where asymmetry is expected could be often indicative of neurological disorder (Toga and Thompson, 2003; Pedraza et al., 2004; Thompson et al., 2007).

The Alzheimer's disease (AD), the most common form of dementia, is a well-known neurodegenerative disorder that presents diffuse lateralized brain atrophies. The same pathological asymmetries were found in the Mild Cognitive Impairment (MCI). Indeed, a large number of morphological-based studies have previously reported a variety of regional abnormalities in hemispheric asymmetry in AD and MCI, including cortical thickness (Kim et al., 2012), cortical volumes (Müller et al., 2005; Pennanen et al., 2005; Shi et al., 2009; Cherbuin et al., 2010; Derflinger et al., 2011; Dhikav et al., 2016; Li et al., 2016), cortical surface area (Thompson et al., 2003, 2007; Long et al., 2013), as well as white matter properties (Müller et al., 2005; Damoiseaux et al., 2009; Stricker et al., 2009; Liu et al., 2011; Wessa et al., 2016; Yang et al., 2017) and functional connectivity (Wang et al., 2015, 2016). The majority of these studies showed that the left hemisphere had a smaller volume than the right, indicating a faster left hemisphere atrophy than in the right side in AD (Thompson et al., 2003, 2007; Müller et al., 2005; Damoiseaux et al., 2009; Li et al., 2016; Wessa et al., 2016).

Among all the brain regions characterized by lateralization, the hippocampus plays a particular and important role as precursor to broader asymmetrical development of the human brain (Pedraza et al., 2004; Woolard and Heckers, 2012; Guadalupe et al., 2014), not only in the general population (Lucarelli et al., 2013), but especially in the Alzheimer's disease (Müller et al., 2005; Shi et al., 2009; Heckemann et al., 2011; Kim et al., 2012; Dhikav et al., 2016). The hippocampus is indeed an essential hub of the neural network of learning and memory, thus any pathological alteration of this region may lead to memory impairment (Squire and Wixted, 2011; Ezzati et al., 2016). A plethora of works showed that AD presented a higher atrophy of left hippocampus respect to the contralateral part and that this asymmetry contributed to cognitive deficits (Müller et al., 2005; Shi et al., 2009; Wicking et al., 2014; Dhikav et al., 2016; Ezzati et al., 2016; Li et al., 2016).

Recently, a growing interest was born on the role of the hippocampal substructures and their relationship with verbal and visual episodic memory in AD (Engvig et al., 2012; Lim et al., 2013; Khan et al., 2015; Zammit et al., 2017), thanks to the improved reliability of their segmentation with *in vivo* MRI techniques (Van Leemput et al., 2009; Iglesias et al., 2015; Whelan et al., 2016). In two previous studies (Vasta et al., 2016; Novellino et al., 2018), our research center already demonstrated the important role of the hippocampal subfields in differentiating AD patients from healthy controls (Vasta et al., 2016), and the strong association of their atrophies with the decline of episodic memory (Novellino et al., 2018). In particular, Vasta et al. (2016) provided the evidence that the measurement of hippocampal subfields represented an advantage over total hippocampal volume for discriminating AD-like phenotypes. Moreover, Novellino et al. (2018) showed for the first time that the *Cornu Ammonis* (CA1, CA4 and dentate gyrus), subiculum and presubiculum hippocampal subfields were selectively involved in AD, and that they were correlated with the mnsesic process.

However, although the lateralized hippocampal atrophy was deeply investigated, few is known about the lateralization of sub-regional hippocampal volumes. Moreover, no one assessed whether and how the asymmetry of hippocampal subfields could be implicated in different memory subsystems, differentially affected in healthy controls, AD and MCI. Thus, the first aim of the present work was to explore the degree of asymmetry in hippocampal substructures as a possible vulnerability biomarker of dementia and of the progression from MCI to AD. For this purpose, we calculated the Asymmetry Index (Pedraza et al., 2004; Heckemann et al., 2011; Kim et al., 2012; Long et al., 2013; Guadalupe et al., 2014; Okada et al., 2016) in a large cohort of healthy controls, AD patients, stable MCI patients and progressive MCI patients from Alzheimer's disease Neuroimaging Initiative database (adni.loni.usc.edu). Our second goal was to evaluate whether significant differences existed in hippocampus substructures between left and right hemisphere in each individual group. Finally, we investigated the relationship between the degree of asymmetry in the substructures of the hippocampus and the decline of memory as assessed by Rey's auditory verbal learning test (Rey, 1964), which is a validated and well-known measure of verbal memory strictly related to hippocampal morphological changes (Estévez-González et al., 2003; Schoenberg et al., 2006; Balthazar et al., 2010; Moradi et al., 2017).

## Materials and methods

### Subjects selection

Data used in the preparation of this article were obtained from the Alzheimer's Disease Neuroimaging Initiative (ADNI) database (adni.loni.usc.edu). The ADNI was launched in 2003 as a public-private partnership, led by Principal Investigator Michael W. Weiner, MD. The primary goal of ADNI has been to test whether serial magnetic resonance imaging (MRI), positron emission tomography (PET), other biological markers, and clinical and neuropsychological assessment can be combined to measure the progression of MCI and early Alzheimer's disease (AD).

We selected publicly available subjects from ADNI by filtering text files downloaded from the website. In particular, we used the file containing the conversion of diagnosis for first choosing 100 healthy controls (HC), 100 Alzheimer's patients (AD) and 100 stable Mild Cognitive Impairment (sMCI) who did not convert their diagnosis in the follow up. With the same approach, we selected 100 progressive MCI who converted to Alzheimer's (pMCI) within 36 months from the baseline.

The second step was to select the visit ID of each subject at the baseline and to obtain demographic, clinical and neuropsychological data at that timepoint, i.e., age, gender, years of education, Mini-Mental State Examination score (MMSE) and Rey's Auditory Verbal Learning Test (RAVLT) scores. The last step was to obtain the subjects' MRI scan id at the baseline from the file MPRAGEMETA.csv. In particular, we selected the first MPRAGE sequence (no repetition), acquired at 3 Tesla.

From the ADNIMERGE table, we extracted the age, gender, years of education, Clinical Dementia Rating Sum of Boxes (CDRSB), Mini Mental State examination (MMSE) and the Rey's auditory verbal learning test (RAVLT) scores.

### RAVLT score

Rey's auditory verbal learning test (RAVLT) (Rey, 1964) is a well-known measure of verbal memory, and its fundamental role in the early diagnosis of AD was established in previous studies (Estévez-González et al., 2003; Schoenberg et al., 2006; Balthazar et al., 2010). Moreover, the strong relationship between the cognitive decline assessed by the RAVLT and the morphological changes in medial temporal lobe structures, especially hippocampus (Wicking et al., 2014), was deeply investigated and demonstrated (Stonnington et al., 2010; Squire and Wixted, 2011). Interestingly, it was shown that in Alzheimer's disease, the RAVLT scores can be predicted from MRI, and among the brain regions, the hippocampus volume resulted to be one the most reliable predictors (Moradi et al., 2017).

Briefly, the RAVLT consists of presenting a list of 15 words across five consecutive trials. The list is read aloud to the participant, and then the participant is immediately asked to recall as many as words as he/she remembers. This procedure is repeated for 5 consecutive trials (Trials 1 to 5). After 30-min of interpolated testing, the participant is again asked to recall the words from the first list (delayed recall).

For each of the 400 subjects that we selected from ADNI, we obtained the RAVLT scores from the ADNIMERGE table. Four different summary scores derived from raw RAVLT scores are provided: the RAVLT Immediate (the sum of scores from 5 first trials, i.e., Trials 1 to 5), the RAVLT Learning (the score of Trial 5 minus the score of Trial 1), the RAVLT Forgetting (the score of Trial 5 minus score of the delayed recall) and RAVLT Percent Forgetting (RAVLT Forgetting divided by the score of Trial 5) (Moradi et al., 2017).

The computing of composite scores, which aggregates several trials, could provide a purer index of specific cognitive processes and it could be considered a better representation of the memory impairment than the raw scores (Vakil et al., 2010). Indeed, the two summary scores, Immediate and Learning, are more informative than the values of the single learning trials, which alone do not reflect the learning process itself. In particular, the RAVLT Immediate reflects the total acquisition/learning, while the RAVLT Learning measures the learning rate (Lezak et al., 2012). Similarly, the other two composite scores, the Forgetting and Percent Forgetting, reflect respectively the long-term retention and forgetting rate by taking into account the Trial 5 as the baseline for the number of words learned, since only the delayed recall score is insufficient for assessing the long-term retention ability (Lezak et al., 2012).

The evidences that RAVLT score is an effective early marker to detect AD in persons with memory complaints (Moradi et al., 2017) lead us to investigate for the first time, whether RAVLT different summary scores could be also be correlated with the degree of magnitude of the hippocampal subfields asymmetry.

### MRI pre-processing

The MRIs were downloaded as raw images converted to the NIFTI format, and then processed by Freesurfer 6.0 with the standard cross-sectional pipeline (*recon-all*). Briefly, the main processing steps of Freesurfer included: removal of non-brain tissue (skull stripping) by using a hybrid watershed/surface deformation procedure (Ségonne et al., 2004), automated Talairach transformation and segmentation of the subcortical white matter and deep gray matter volumetric structures (including hippocampus, amygdala, caudate, putamen, and ventricles) (Fischl et al., 2004). The estimated total intracranial volume (ICV) was also calculated. More technical details of these procedures were described in previous publications (Dale et al., 1999; Fischl et al., 2002, 2004; Ségonne et al., 2004).

Moreover, with Freesurfer 6.0, we performed a reliable automated segmentation of the hippocampus to its respective subfields (Whelan et al., 2016) by using Bayesian inference and a probabilistic atlas of the hippocampal formation based on manual delineations of subfields in ultra-high T1-weighted MRI scans from a number of different training subjects (Van Leemput et al., 2009; Iglesias et al., 2015). The hippocampus was anatomically divided into: parasubiculum, presubiculum, subiculum, CA1, CA3, CA4 (cornu ammonis areas), granule cells in the molecular layer of the dentate gyrus (GC-ML-DG), hippocampal-amygdaloid transition area (HATA), fimbria, molecular layer, hippocampal fissure and hippocampal tail. Segmentation results were also visually inspected by an expert neurologist (F.N.) for errors, but no manual edits were needed.

### Asymmetry index

The magnitude of asymmetry of the hippocampus and its subfields was calculated by taking the percentage ratio between the absolute value of the difference between left and right raw volume (not normalized by ICV) and the sum of them (Pedraza et al., 2004; Heckemann et al., 2011; Kim et al., 2012; Long et al., 2013; Guadalupe et al., 2014; Okada et al., 2016), as follow:

$$\begin{array}{l} \text{AsymmetryIndex},\;\text{AI}\;=\;\frac{\left|Left-Right\right|}{Left+Right}*100, \end{array}$$

where lower AI values indicated a decrease of the degree of asymmetry in the specific structure, i.e., AI = 0 when Left = Right. Thus, in this work, the AI represents an asymmetry measure not directed toward one particular hemisphere, on the opposite of the lateralization index (Derflinger et al., 2011). It is worth of noting that, the AI corrects for overall hippocampal volume and for the subfields' volumes (Galaburda et al., 1987; Woolard and Heckers, 2012), thus taking into account for the variability in region size.

### Statistical analysis

The statistical analysis was conducted by using the R language 3.3.2 for Macintosh. An analysis of variance (ANOVA) was employed for comparing the age, the years of education, the CDRBS, the MMSE and the RAVLT scores among the four groups. The multiple comparisons problem was accounted by a *post-hoc* Tukey's honest significant difference (HSD) test (*p* < 0.05). Differences in the gender distribution were assessed with a Pearson Chi-square test (*p* < 0.05).

An analysis of covariance (ANCOVA) was applied for finding differences in each of the hippocampal subfields AIs among the four groups, by adding age, gender, years of education and ICV as covariates, as AIs might be not distributed normally (Kim et al., 2012; Long et al., 2013; Okada et al., 2016). The significance level for the *post-hoc* paired comparisons was adjusted by the Tukey's HSD test (p < 0.05).

With the aim of exploring the possible lateralization of the hippocampal subfields, a paired *t*-test was used for comparing left and right normalized volumes of these regions, separately for each of the four groups (p < 0.05).

Partial correlation, controlling for age, gender, years of education and ICV, was used for exploring the relationship between the neuropsychological tests and the AI values of the hippocampal subfields. In particular, we separately regressed out from the RAVLT scores and the AI values the influence of age, gender, years of education and ICV with a linear regression. Then, we performed a Pearson's correlation between the residuals of RAVLT scores and residuals of AIs.

In paired *t*-tests, ANCOVAs and partial correlations, the statistical threshold of the *post-hoc* analyses corrected according to Bonferroni: *p* < 0.05/13 = 0.0038, considering 13 comparisons (the whole hippocampus and its 12 substructures).

## Results

### Subjects characteristics

Table 1 summarized demographics, clinical and neuropsychological characteristics of the four groups at the baseline. ANOVA did not reveal any differences among groups in age and years of education, and no differences in gender distribution was found by pairwise Chi-square tests.

**Table 1:** Demographic, clinical, and neuropsychological characteristics of the four groups at the baseline, and results of statistical comparisons.

	**HC (*n* = 100)**	**sMCI (*n* = 100)**	**pMCI (*n* = 100)**	**AD (*n* = 100)**	**ANOVA *p*-value**	***Post-hoc* (*p* < 0.05)**
Age	73.36 ± 5.76	72.27 ± 7.71	72.47 ± 6.89	74.14 ± 7.72	0.219	N.S.
Female (%)	52%	49%	50%	48%	*N*.*A*.	N.S.^§^
Education (yrs)	16.38 ± 2.55	16.38 ± 2.69	15.9 ± 2.84	15.73 ± 2.78	0.221	N.S.
CDRSB	0.06 ± 0.28	1.22 ± 0.72	2.21 ± 1.02	4.32 ± 1.65	< 0.001^*^	HC < sMCI HC < pMCI HC < AD sMCI < pMCI sMCI < AD pMCI < AD
MMSE	29.09 ± 1.11	28.05 ± 1.72	27.34 ± 1.86	23.13 ± 2.10	< 0.001^*^	HC>sMCI HC>pMCI HC>AD sMCI>pMCI sMCI>AD pMCI>AD
RAVLT Immediate	45.40 ± 10.29	39.80 ± 10.51	29.08 ± 7.72	23.08 ± 7.84	< 0.001^*^	HC>sMCI HC>pMCI HC>AD sMCI>pMCI sMCI>AD pMCI>AD
RAVLT Learning	5.75 ± 2.39	5.17 ± 2.61	3.23 ± 2.48	1.70 ± 1.62	< 0.001^*^	HC>pMCI HC>AD sMCI>pMCI sMCI>AD pMCI>AD
RAVLT Forgetting	3.92 ± 2.79	3.9 ± 2.38	5.25 ± 2.23	4.29 ± 1.84	< 0.001^*^	HC < pMCI HC < AD sMCI < pMCI pMCI>AD
RAVLT Percent forgetting	37.06 ± 28.87	44.36 ± 29.82	76.31 ± 25.26	86.09 ± 26.61	< 0.001^*^	HC < pMCI HC < AD sMCI < pMCI sMCI < AD

*Data are given as mean ± SD*.

* *ANOVA among groups significant at p < 0.05*.

§ *Not significant at pairwise Chi-square tests. Abbreviation: yrs, years; CDRSB, Clinical Dementia Rating Sum of Boxes; MMSE, Mini Mental State Examination; HC, healthy control; MCI, Mild Cognitive Impairment; sMCI, stable MCI; pMCI, progressive MCI; AD, Alzheimer's disease; N.A., not applicable; N.S., Not significant*.

*Post-hoc* analysis revealed that the MMSE, the CDRSB and the RAVLT Immediate scores were significantly different in all the pairwise comparisons with a RAVLT progressive score reduction from demented to not demented patients.

Regarding the RAVLT Learning, the mean values were significantly different among groups, except that for the comparison between HC and sMCI. The mean values of RAVLT forgetting were significantly different among groups, except that for the comparison between HC and sMCI and sMCI and AD. Finally, the mean values of RAVLT Percent Forgetting were significantly different among groups, except that for the comparison between HC and sMCI and pMCI and AD.

### Hippocampal subfields asymmetry

**Figure 1:**
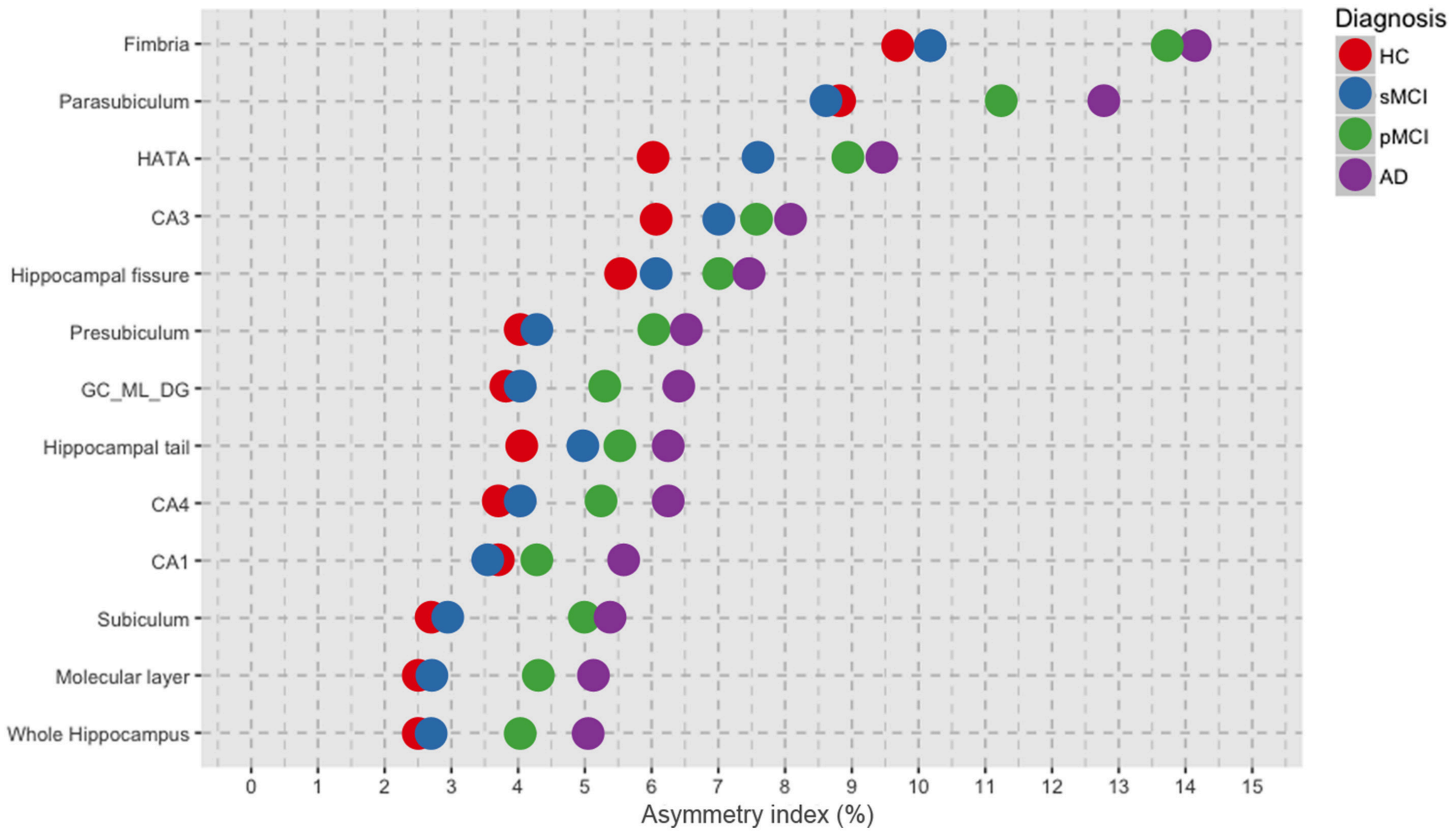
Plot of the mean values per group of asymmetry index for each of the 12 hippocampal subfields and for the whole hippocampus, ordered by decreasing magnitude of asymmetry. HC, healthy control; MCI, Mild Cognitive Impairment; sMCI, stable MCI; pMCI, progressive MCI; AD, Alzheimer's disease.

The asymmetry means values of the hippocampal subfields and of the whole hippocampus are reported by group in Table 2. Moreover, AIs ordered by decreasing values depicted in Figure 1 revealed a general trend of increased asymmetry with the increased severity of diagnosis. Indeed, AD had the higher magnitude of asymmetry in all of the 12 hippocampal subfields and in the whole hippocampus.

**Table 2:** Asymmetry index mean values of hippocampal subfields for each group and results of ANCOVA and *post-hoc* comparisons survived at Bonferroni's correction (*p* < 0.0038).

**Asymmetry Index**	**HC**	**sMCI**	**pMCI**	**AD**	**ANCOVA *p-value***	***Post-hoc (p < 0.0038)***
Hippocampus	2.54 ± 2.17	2.67 ± 2.59	4.03 ± 3.89	5.13 ± 4.29	< 0.001^*^	HC < AD sMCI < AD
Parasubiculum	8.77 ± 6.12	8.62 ± 7.00	11.20 ± 8.44	12.70 ± 9.59	0.001^*^	N.S.
Presubiculum	4.04 ± 2.99	4.33 ± 3.71	6.12 ± 5.05	6.49 ± 5.78	< 0.001^*^	HC < AD
Subiculum	2.87 ± 2.54	2.98 ± 2.64	4.90 ± 4.82	5.49 ± 5.05	< 0.001^*^	HC < pMCI HC < AD sMCI < AD
CA1	3.69 ± 2.99	3.62 ± 2.75	4.27 ± 4.20	5.54 ± 4.71	0.002*	N.S.
CA3	6.27 ± 4.36	7.19 ± 4.7	7.59 ± 5.12	8.04 ± 6.26	0.108	N.S.
CA4	3.66 ± 2.85	4.00 ± 3.05	5.33 ± 3.96	6.35 ± 4.75	< 0.001^*^	HC < AD sMCI < AD
GC-ML-DG	3.8 ± 2.95	4.00 ± 3.05	5.33 ± 3.95	6.40 ± 4.95	< 0.001^*^	HC < AD sMCI < AD
HATA	6.03 ± 4.31	7.52 ± 6.53	8.96 ± 7.59	9.45 ± 8.67	0.006^*^	N.S.
Fimbria	9.68 ± 8.84	10.2 ± 11.31	13.72 ± 10.39	14.12 ± 11.18	0.003^*^	N.S.
Molecular layer	2.53 ± 2.13	2.66 ± 2.57	4.31 ± 4.27	5.29 ± 4.51	< 0.001^*^	HC < pMCI HC < AD sMCI < AD
Hippocampal fissure	5.43 ± 4.73	6.21 ± 4.71	7.24 ± 6.41	7.46 ± 6.13	0.033^*^	N.S.
Hippocampal tail	4.28 ± 3.03	4.98 ± 3.83	5.49 ± 5.64	6.36 ± 4.99	0.019^*^	N.S.

*Data are given as mean ± SD*.

* *ANCOVA among groups significant at p < 0.05. Abbreviation: HC, healthy control; MCI, Mild Cognitive Impairment; sMCI, stable MCI; pMCI, progressive MCI; AD, Alzheimer's disease; N.S., Not significant*.

**Figure 2:**
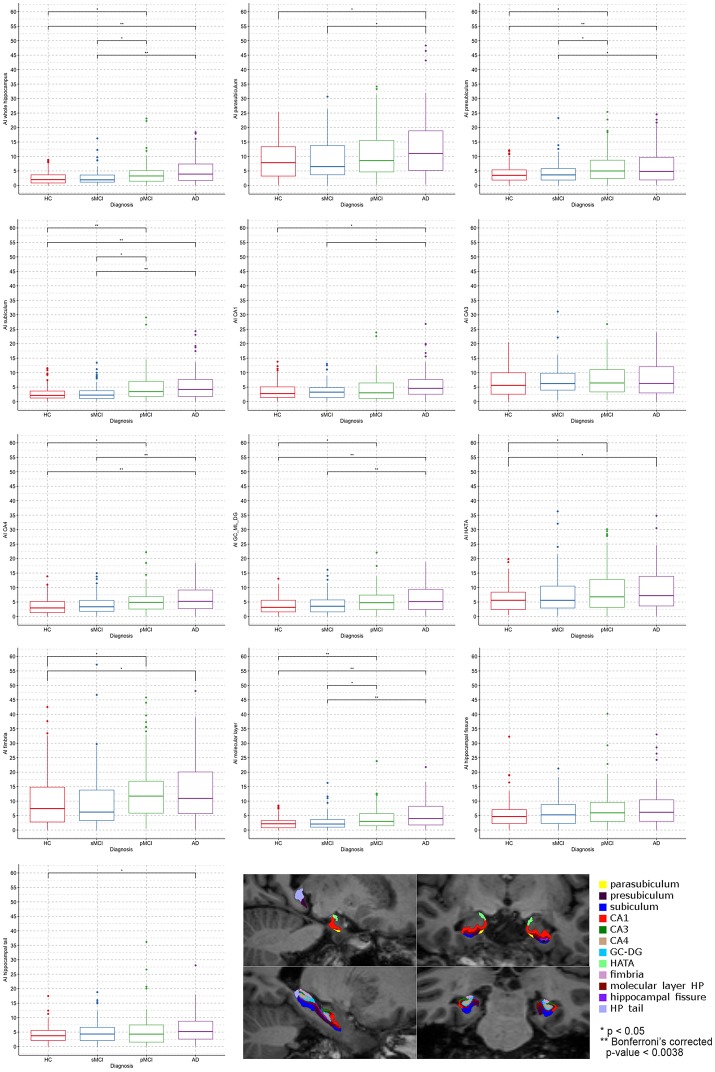
Boxplots by diagnosis of the asymmetry mean values of the whole hippocampus and its 12 subfields. **Pairwise comparison survived at Bonferroni's correction (*p* < 0.0038). In the bottom right corner is an example of hippocampal subfields segmentation of a healthy control performed by Freesurfer 6.0.

ANCOVA results (box plots reported in Figure 2) showed that the magnitude of asymmetry of the whole hippocampus was significantly different at *post-hoc* analyses and survived at Bonferroni's correction, between HC and AD, and between sMCI and AD. Significant differences in the asymmetry index of presubiculum were found only between HC and AD. The asymmetry of subiculum was different between HC and pMCI, HC and AD, and sMCI and AD. CA4 and GC-ML-DG showed differences in their asymmetry index only between HC and AD, and sMCI and AD. The molecular layer presented significant differences in the degree of asymmetry between HC and pMCI, HC and AD, and sMCI and AD. None of the pairwise comparisons survived at Bonferroni's correction in the parasubiculum, CA1, CA3, HATA, fimbria, hippocampal fissure and hippocampal tail. For the sake of knowledge, the boxplots in Figure 2 also reported significant *post-hoc* comparisons at *p* < 0.05.

**Figure 3:**
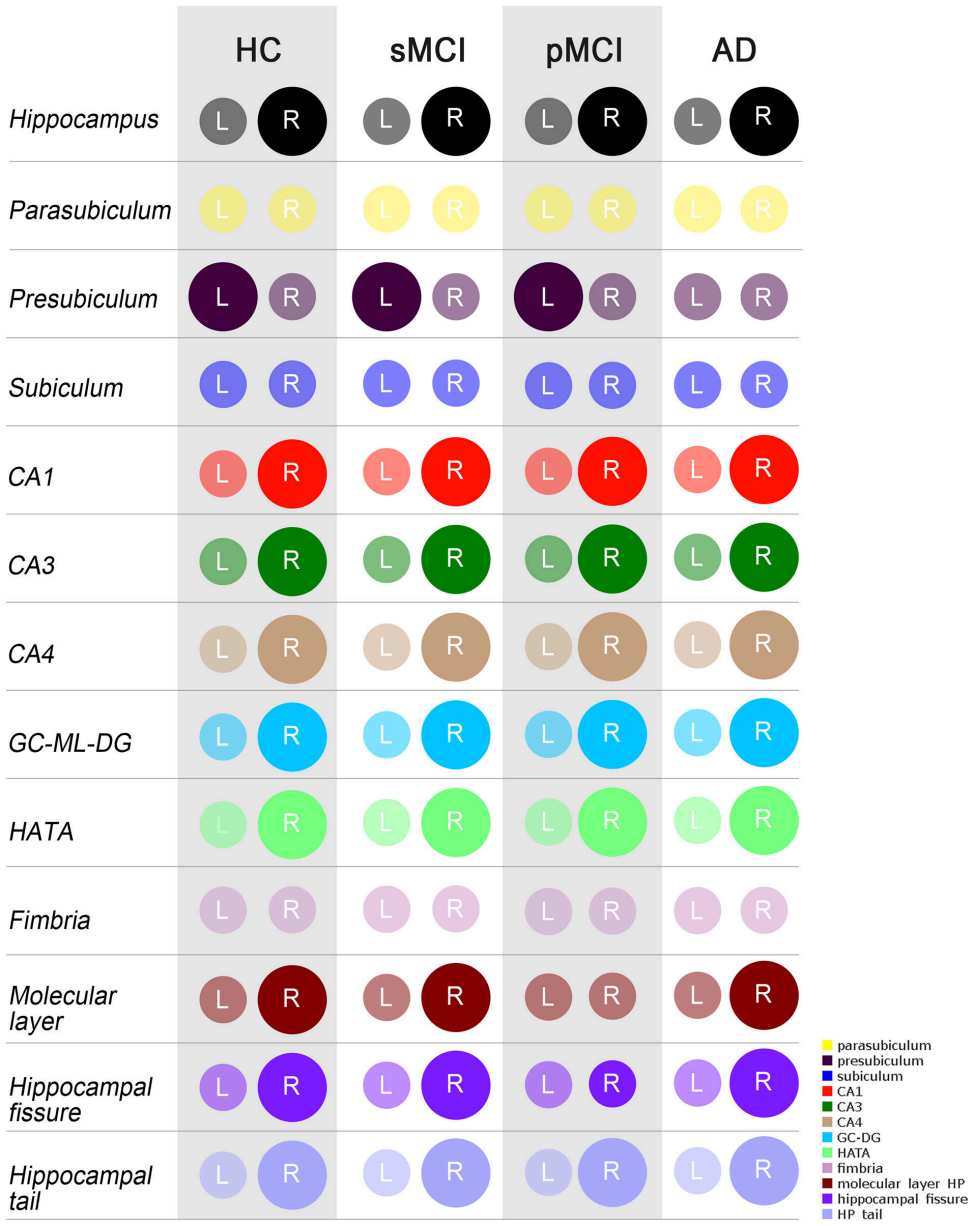
Schematic representation of significant differences as evaluated by the paired *t*-test (Bonferroni's correction) between left and right volumes, normalized by ICV, of the whole hippocampus and its 12 subfields. A larger diameter of the circle indicated a larger volume in the respective hemisphere. Colors are taken from the original legend provided by Freesurfer 6.0 (in the bottom right corner). HC, healthy control; MCI, Mild Cognitive Impairment; sMCI, stable MCI; pMCI, progressive MCI; AD, Alzheimer's disease; L, left; R, right.

ANOVA did not reveal any differences among groups in intracranial volume. In Table 3, the mean raw volumes of hippocampus and its twelve subfields are reported, together with the *p*-values of the paired *t*-tests, which compared left and right volumes for each individual group normalized by the ICV (also reported in Table 3). Figure 3 depicted a schematic representation of the significant (survived at Bonferroni's correction) lateralizations found in the hippocampal subfields, that is which hemisphere had a higher volume respect to its contralateral part. All the groups showed rightward lateralization (right > left) in the whole hippocampus, CA1, CA3, CA4, GC-ML-DG, HATA, hippocampal fissure and hippocampal tail. A leftward lateralization (left > right) was found in the presubiculum of HC, sMCI and pMCI, but not in AD. No significant differences between left and right volumes were found in the parasubiculum, subiculum, and fimbria. Regarding the molecular layer, HC, sMCI and AD presented a rightward lateralization, while pMCI did not present any lateralization in this subfield.

**Table 3:** Mean raw volumes (not normalized for ICV) of hippocampus and its twelve subfields for each group, and results of paired *t*-tests on ICV normalized volumes.

**Volumes [mm^3^]**	**HC**			**sMCI**			**pMCI**			**AD**		
	**Left**	**Right**	***p*-value**	**Left**	**Right**	***p*-value**	**Left**	**Right**	***p*-value**	**Left**	**Right**	***p*-value**
Hippocampus	3155.56 ± 414.09	3277.13 ± 404.10	< 0.001^*^	3019.87 ± 455.15	3152.01 ± 450.50	< 0.001^*^	2714.70 ± 443.43	2819.08 ± 435.51	< 0.001^*^	2492.85 ± 421.49	2646.59 ± 427.67	< 0.001^*^
Parasubiculum	60.32 ± 14.16	60.78 ± 11.18	0.55	58.68 ± 13.52	58.97 ± 13.99	0.94	57.63 ± 15.92	54.31 ± 14.84	0.025	51.80 ± 15.11	52.19 ± 14.32	0.72
Presubiculum	296.10 ± 44.55	284.82 ± 42.20	< 0.001^*^	281.27 ± 49.37	269.30 ± 44.12	< 0.001^*^	250.96 ± 51.08	235.71 ± 40.54	< 0.001^*^	226.26 ± 49.91	221.03 ± 44.53	0.18
Subiculum	403.76 ± 57.15	411.84 ± 56.50	0.0089	387.90 ± 65.30	388.43 ± 57.15	0.88	340.20 ± 63.69	341.76 ± 60.09	0.72	309.68 ± 62.58	319.71 ± 60.64	0.026
CA1	585.53 ± 82.61	619.73 ± 89.98	< 0.001^*^	563.85 ± 89.09	595.30 ± 96.87	< 0.001^*^	516.33 ± 89.34	540.31 ± 90.45	< 0.001^*^	479.11 ± 86.49	506.50 ± 87.46	< 0.001^*^
CA3	183.76 ± 31.08	203.44 ± 34.57	< 0.001^*^	180.12 ± 33.83	202.89 ± 35.02	< 0.001^*^	162.75 ± 31.44	183.11 ± 35.62	< 0.001^*^	153.08 ± 28.55	175.75 ± 36.92	< 0.001^*^
CA4	229.02 ± 32.24	239.19 ± 31.59	< 0.001^*^	221.80 ± 34.46	235.42 ± 34.56	< 0.001^*^	199.57 ± 32.56	214.21 ± 35.75	< 0.001^*^	185.46 ± 32.39	203.21 ± 33.32	< 0.001^*^
GC-ML-DG	263.60 ± 38.44	276.46 ± 38.75	< 0.001^*^	254.57 ± 41.17	270.45 ± 41.49	< 0.001^*^	227.38 ± 39.19	243.69 ± 41.76	< 0.001^*^	211.13 ± 38.64	230.62 ± 39.46	< 0.001^*^
HATA	56.48 ± 9.90	61.05 ± 10.59	< 0.001^*^	53.13 ± 10.40	58.70 ± 11.40	< 0.001^*^	47.08 ± 10.99	50.91 ± 10.06	< 0.001^*^	44.06 ± 10.75	49.34 ± 10.30	< 0.001^*^
Fimbria	73.18 ± 24.27	71.27 ± 24.17	0.32	68.20 ± 23.72	68.57 ± 20.17	0.85	57.11 ± 25.12	55.78 ± 23.95	0.48	51.72 ± 21.83	51.98 ± 20.10	0.88
Molecular layer	514.77 ± 68.82	533.41 ± 68.17	< 0.001^*^	493.58 ± 77.87	511.25 ± 76.78	< 0.001^*^	440.48 ± 76.15	453.70 ± 74.21	0.0072	401.64 ± 72.46	424.47 ± 73.14	< 0.001^*^
Hippocampal fissure	163.02 ± 31.64	173.32 ± 30.05	< 0.001^*^	162.09 ± 29.72	172.07 ± 30.40	< 0.001^*^	163.96 ± 33.48	171.94 ± 36.28	0.0084	152.96 ± 30.66	165.07 ± 33.44	< 0.001^*^
Hippocampal tail	489.04 ± 79.84	515.15 ± 78.58	< 0.001^*^	456.74 ± 82.87	492.70 ± 89.57	< 0.001^*^	415.21 ± 77.70	445.59 ± 71.55	< 0.001^*^	378.90 ± 68.80	411.76 ± 74.15	< 0.001^*^
ICV [liters]	1.52 ±, 0.19			1.53 ± 0.17			1.53 ± 0.19			1.52 ± 0.19		

*Data are given as mean ± SD*.

* *Paired t-test on ICV normalized volumes is significant at Bonferroni's correction (p < 0.0038). Abbreviation: HC, healthy control; MCI, Mild Cognitive Impairment; sMCI, stable MCI; pMCI, progressive MCI; AD, Alzheimer's disease; ICV, Intracranial volume*.

### Correlation between asymmetry index and neuropsychological scores

**Figure 4:**
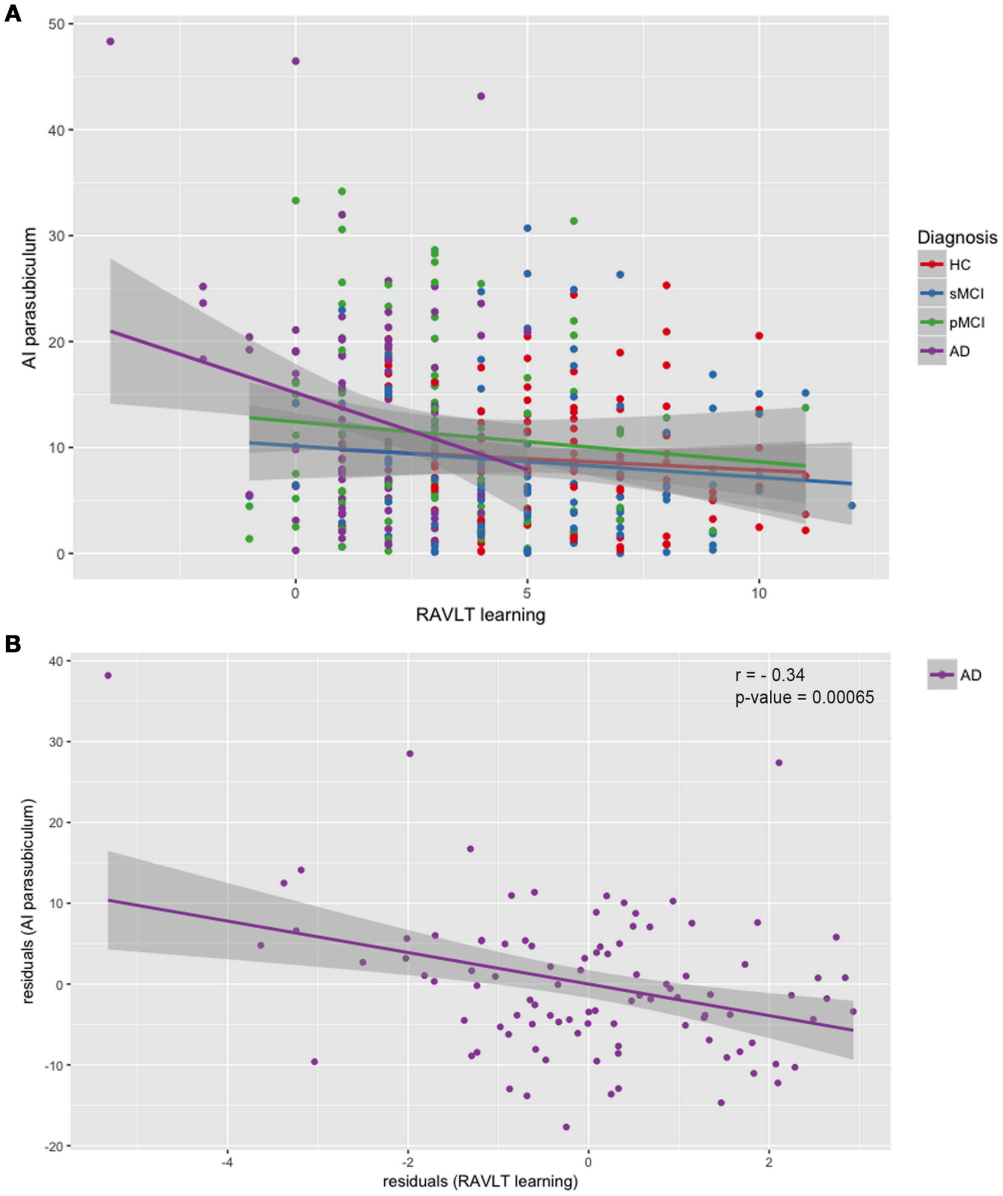
Scatterplots of asymmetry index of the parasubiculum and RAVLT Learning scores. **(A)** Linear regression performed separately for each diagnosis on raw data; **(B)** Linear regression and partial correlation performed on the residuals of AI of the parasubiculum and the residuals of the RAVLT Learning scores, obtained by partialling-out the effect of age, gender, years of education and intracranial volume. Correlation coefficient *r* = −0.33, *p* = 0.00065. HC, healthy control; MCI, Mild Cognitive Impairment; sMCI, stable MCI; pMCI, progressive MCI; AD, Alzheimer's disease; AI, asymmetry index.

We performed a partial correlation between the asymmetry indices and the MMSE score, and the RAVLT Immediate, Learning, Forgetting and Percent Forgetting scores after covarying-out the influence of age, gender, years of education and intracranial volume in each of the four groups. The only significant relationship that survived at Bonferroni's correction was found in AD between the AI of the parasubiculum and the RAVLT Learning, with a negative correlation coefficient r of −0.33 and a *p*-value of 0.00065. The not significant results of the partial correlation between the RAVLT Learning and the AI of the parasubiculum for the other groups were: HC: *r* = −0.09, *p* = 0.361; sMCI: *r* = 0.03, *p* = 0 0.772; pMCI: *r* = −0.11, *p* = 0.266. In Figure 4A, we reported the scatterplot of raw values (not adjusted for the covariates) of the parasubiculum AI and the RAVLT Learning scores, by adding the regression line for each diagnosis. In Figure 4B, we reported the scatterplot of the same data but regressed out from the covariates, i.e., the residuals, of this only significant relationship between the two variables in Alzheimer's patients.

## Discussion

In the current work, we investigated for the first time the MRI asymmetry and lateralization of hippocampal subfields volumes in the AD and MCI. We found an increment of the magnitude of asymmetry with the increase of the severity of the diagnosis in all the hippocampal subfields, thus through the continuum from the healthy subjects to AD, through sMCI and pMCI. We also confirmed that the Alzheimer's disease presented a strong leftward atrophy of the whole hippocampus, but for the first time we demonstrated that the hippocampal subfields had different lateralizations compared to the whole region. Furthermore, our findings were strengthened by the relationship between the degree of asymmetry of parasubiculum and the learning rate in AD patients.

Brain asymmetry is considered a critical morphological metric reflecting evolutionary, developmental and pathological changes of the human brain. In particular, the existence of asymmetry in some specific brain regions could be often indicative of neurological disorder (Wolf et al., 2001; Toga and Thompson, 2003). Generally, the brain atrophy of AD was described as a spread process with no hemispheric predilection (Derflinger et al., 2011). However, early neuroimaging studies investigating AD have observed strong differences between the left and right side of the ventricles or in the anterolateral temporal cortex, thus demonstrating that disease progresses asymmetrically (Toga and Thompson, 2003; Shi et al., 2009; Heckemann et al., 2011). Moreover, the degree of leftward atrophy of hippocampus resulted to increase with the disease progression, thus suggesting that this variation might characterize the onset of AD (Pedraza et al., 2004; Cherbuin et al., 2010; Heckemann et al., 2011; Woolard and Heckers, 2012; Lee et al., 2017). In this work, we provided new evidence that the hippocampal subfields present different degree of asymmetry. In particular, significant differences in the AI were detected between AD with respect to HC and sMCI patients in the whole hippocampus, subiculum, CA4, GC-ML-DG and molecular layer, whilst in the pMCI group we found an intermediate magnitude of asymmetry, placing in a sort of continuum between sMCI and AD. The presence of morphological signs between pMCI and sMCI represents a critical point for ascertaining the biological basis of clinical conversion to dementia. At a volumetric level, several works had demonstrated the utility of the hippocampal subfields in discriminating patients individually. Indeed, Khan et al. (2015) found that combined subfield volume and presubiculum volume were more accurate than total hippocampal volume in predicting the conversion to AD. Vasta et al. (2016) confirmed the results of Khan et al. (2015) showing that the subiculum and presubiculum together had a higher specificity than the whole hippocampus in distinguishing sMCI from pMCI. In the present work, we sought to demonstrate whether the AI of the hippocampal subregions could characterize the progression to AD. Although a trend toward differences between sMCI and pMCI in AI was detected in the subiculum, presubiculum, and molecular layer, this did not survive at Bonferroni's correction. Thus, we cannot clearly conclude that the AI of hippocampal subfields represent a marker of progression to AD. For this reason, further studies using multivariate statistical analysis (i.e., Machine Learning approaches) must be performed in order to understand which specific combination of asymmetry-related morphological features could be used to discriminate at an individual level, stable from progressive MCI patients.

When we considered the directionality of the asymmetries, we found a general and common trend of lateralization among HC, sMCI, pMCI, and AD toward the right hemisphere (right volume > left volume) of eight on twelve hippocampal substructures—CA1, CA3 CA4, GC-ML-DG, HATA, molecular layer, hippocampal fissure and hippocampal tail—as well as in the whole hippocampus, in the four diagnostic groups. Three hippocampal subfields did not present any significant lateralization toward a specific hemisphere in the four groups: the parasubiculum, subiculum, and fimbria. The presubiculum showed a leftward lateralization (left volume > right volume) in healthy controls, stable and progressive MCI, but not in AD, which did not present any significant difference between left and right hemisphere in this substructure. The molecular layer was another hippocampal subfield that did not present a homogeneous pattern of lateralization among our four groups. Even if in HC, sMCI and AD, the molecular layer showed a higher volume in the right hemisphere, in pMCI no statistical differences were found between the right and left hemisphere. The consistent left-less-than-right asymmetry of the whole hippocampus volume in normal subjects, MCI and AD patients, was largely demonstrated by several works summarized in a meta-analysis by Shi et al. (2009). Our results corroborated the existence of the leftward atrophy of the whole hippocampus in AD and MCI. The origin of this left hippocampal susceptibility is still unknown and it had been hypothesized that it could be related to hormonal, genetic as well as development and environmental factors (Toga and Thompson, 2003; Woolard and Heckers, 2012). Another explanation is that the left hippocampus is more vulnerable to AD pathology since in healthy subjects it is physiologically smaller than the right one (Müller et al., 2005), as observed also in our controls. The anatomical and functional asymmetry from anterior to posterior structure of the hippocampus was already documented by Woolard and Heckers (2012). In the current work—for the first time—we demonstrated that also the hippocampal subfields had distinct patterns of volume lateralization, which were different compared to the whole region.

An interesting result presented in the current work regards the hemispheric alterations of the parasubiculum and its relationship with the verbal memory. Despite the lack of interhemisferic volumetric difference, the AD was the unique group showing a significant correlation between AI and RAVLT learning scores. We found that the more asymmetric was the parasubiculum, the worst was the performance at the rate of learning. It is well known that the decline of hemispheric asymmetry during healthy aging and disease progress represents an impediment to the compensation and interaction between the hemispheres, which badly influence the cognitive functions (Long et al., 2013). Woolard and Heckers (2012) found a relationship between the degree of right > left asymmetry of hippocampus and the performance on the verbal learning, verbal fluency and motor-processing speed. One more study (Wolf et al., 2001) found that the magnitude of non-directional hippocampal asymmetry increased with decreasing cognitive state. Although numerous works investigated the association between hippocampal volume and neurocognitive tests, the relationships between hippocampal subfields and neurocognitive measures were poorly studied. Lim et al. (2012), reported associations between atrophy in the CA1 and subiculum and verbal immediate recall, verbal delayed recall, verbal recognition memory, and constructional recall. Zammit et al. (2017) confirmed that CA1 and subiculum had a specific role in episodic memory and one recent work of our research group (Novellino et al., 2018) demonstrated for the first time with a multimodal analysis that the subiculum, CA1 and CA4-DG were correlated with immediate and delayed total recall items of Free and Cued Selective-Reminding-Test (FCSRT). Surprisingly, the negative association that we discovered between the AI of parasubiculum and the RAVLT Learning results is an actual novelty. The potential reason for different results observed between previous studies and our study, is probably linked to the different measures employed. Indeed, it is the first time that an asymmetry index—rather than the volume of hippocampal subfields – was correlated with the memory outcomes. The parasubiculum together with the presubiculum are considered the main hub for the complex hippocampal memory system (Dalton and Maguire, 2017). In particular, the parasubiculum receives strong inputs from the anterior thalamic nuclei, forming a pathway that, when disconnected, can affect hippocampal processing of incoming information (Byrne, 2010). Thus, we can argue that the rate of learning, rather than the total acquisition/learning performance, was negatively affected by the asymmetrical but not lateralized atrophies of the parasubiculum, which caused the impairment of memory and learning flexibility. In other words, we can speculate that in normal brain, the parasubiculum is bilaterally involved in the rapid acquisition of new information and that when, indifferently the left or right hemisphere has higher atrophy than its contralateral, the learning rate slows down as in AD patients. However, this explanation will remain only an intriguing hypothesis until new evidence will confirm the relationship between the parasubiculum and the learning process, as assessed by other specific memory batteries. Indeed, a further study could assess the long-term variations of the asymmetry of hippocampal subfields and their impact on the learning and remembering of new information in the normal aging as well in neurodegenerative diseases.

Some important caveats need to be discussed. Firstly, regarding the automatic segmentation of the hippocampal subfields (Van Leemput et al., 2009; Iglesias et al., 2015), it should be reported that only recently Freesurfer 6.0 produced four new substructures (parasubiculum, molecular layer, granule cells of dentate and HATA). However, despite the novelty of this approach, the high heritability and reliability of the automatic segmentation of the hippocampal formation subregions by Freesurfer 6.0 was demonstrated by Whelan et al. (2016), also when data were collected and analyzed at multiple, independent sites as for the ADNI project. Secondly, further research could explore the sensitivity of different asymmetry indices in hippocampal substructures. Indeed at least three main types of asymmetry exist, each characterized by a different combination of mean and variance of the distribution (Palmer and Strobeck, 1986). We used the absolute values of the ratio between the difference of left and right volume and the sum of the hemispheres volumes, which is a widely accepted and applied measure of the MRI magnitude of asymmetry (Pedraza et al., 2004; Heckemann et al., 2011; Kim et al., 2012; Long et al., 2013; Guadalupe et al., 2014; Okada et al., 2016). However, the discriminatory diagnostic ability of this particular index should be examined more closely by a comparison with other kind of asymmetry measures as reported by Palmer and Strobeck (1986).

In conclusion, our methodology showed that the hippocampal substructures exhibited different sub-patterns of asymmetry and lateralization compared to the whole hippocampus, through the continuum from healthy subjects to MCI and AD. Moreover, we found an increment of the magnitude of asymmetry with the increment of the severity of the diagnosis, where AD patients had the highest values of asymmetry in all the hippocampal subfields as well as in the whole hippocampus. More interestingly, the pathological low rate of learning was correlated with the pathological high degree of asymmetry in the parasubiculum of AD patients, indicating the possibility of using AI as a biomarker for the disease progression.

## Author contributions

AS: Research project—Conception, Organization, and Execution; Statistical Analysis—Design, Execution, Review and Critique; Manuscript—Writing of the first draft, Review and Critique. RV: Research project—Organization and Execution; Statistical Analysis—Design, Review and Critique; Manuscript—Review and Critique. FN: Research project—Conception, Organization and Execution; Manuscript—Review and Critique. MGV: Research project—Organization; Manuscript—Review and Critique. AC: Research project—Organization and Execution; Manuscript—Review and Critique. AQ: Research project—Organization and Execution; Manuscript—Review and Critique.

### Conflict of interest statement

The authors declare that the research was conducted in the absence of any commercial or financial relationships that could be construed as a potential conflict of interest.
